# Two identified looming detectors in the locust: ubiquitous lateral connections among their inputs contribute to selective responses to looming objects

**DOI:** 10.1038/srep35525

**Published:** 2016-10-24

**Authors:** F. Claire Rind, Stefan Wernitznig, Peter Pölt, Armin Zankel, Daniel Gütl, Julieta Sztarker, Gerd Leitinger

**Affiliations:** 1 Institute of Neuroscience/Centre for Behaviour and Evolution, Newcastle University, Newcastle upon Tyne, NE1 7RU, UK; 2 Institute of Cell Biology, Histology and Embryology/Research Unit Electron Microscopic Techniques, 8010 Graz, Austria; 3 Institute for Electron Microscopy and Nanoanalysis/NAWI Graz, Graz University of Technology, 8010 Graz, Austria; 4 Centre for Electron Microscopy, 8010 Graz, Austria; 5 Departamento de Fisiologıa, Biologıa Molecular y Celular/FCEN, Universidad de Buenos Aires/IFIBYNE-CONICET, Buenos Aires 1428, Argentina; 6 BioTechMed-Graz, 8010 Graz, Austria

## Abstract

In locusts, two lobula giant movement detector neurons (LGMDs) act as looming object detectors. Their reproducible responses to looming and their ethological significance makes them models for single neuron computation. But there is no comprehensive picture of the neurons that connect directly to each LGMD. We used high-through-put serial block-face scanning-electron-microscopy to reconstruct the network of input-synapses onto the LGMDs over spatial scales ranging from single synapses and small circuits, up to dendritic branches and total excitatory input. Reconstructions reveal that many trans-medullary-afferents (TmAs) connect the eye with each LGMD, one TmA per facet per LGMD. But when a TmA synapses with an LGMD it also connects laterally with another TmA. These inter-TmA synapses are always reciprocal. Total excitatory input to the LGMD 1 and 2 comes from 131,000 and 186,000 synapses reaching densities of 3.1 and 2.6 synapses per μm^2^ respectively. We explored the computational consequences of reciprocal synapses between each TmA and 6 others from neighbouring columns. Since any lateral interactions between LGMD inputs have always been inhibitory we may assume these reciprocal lateral connections are most likely inhibitory. Such reciprocal inhibitory synapses increased the LGMD’s selectivity for looming over passing objects, particularly at the beginning of object approach.

A powerful concept is that of “feature-detection,” where the nervous system filters natural stimuli to selectively respond to things of biological significance^1^,^2^ and insect visual systems provide some well-known examples^3^,^4^,^5^. In the locust two large neurons, the Lobula Giant Movement Detectors LGMD 1 and 2, detect the image changes caused by looming objects^6^. Minutes after a locust emerges from the egg, they recognize looming objects and can co-ordinate appropriate escape behaviors that enable the locust to escape and survive^7^,^8^,^9^. The timing of the peak response of the LGMDs remains remarkably consistent even when looming occurs against a moving background^6^,^10^. Due to the reproducibility of the LGMD 1’s response to looming and its ethological significance^11^,^12^,^13^ the LGMD 1 is considered a model system for single neuron computation^14^,^15^.

However we know comparatively little about the contribution to looming object detection of the precise organization of inputs directly onto the LGMDs. A recent model which successfully reproduces the timing of the peak of the LGMD response to looming stimuli does so using simple addition of presynaptic inputs by the LGMD 1^16^,^17^. This may not be the entire picture because the LGMD does more than respond with a peak to looming objects, it can signal approach against a moving background and it ignores translating objects^5^,^6^,^10^,^18^,^19^,^20^,^21^. For this wider range of responses, a feature of the input organization of the LGMD 1 has been shown to be important, that of laterally spreading inhibition^18^,^22^,^23^. Lateral inhibition explains why the LGMD 1 excitatory response to motion of a small dark object measured experimentally in the dendrites of the LGMD 1, is suppressed by motion of another object within 3.8 degrees of the first, or by a series of moving features such as a moving sinewave grating or a striped pattern. Lateral inhibition occurs presynaptically to the LGMD because no inhibitory postsynaptic potentials (PSPs) are recorded intracellularly in the LGMD 1 during this response suppression, even when the LGMD 1 is hyperpolarized to reveal any silent PSPs that could have reversal potentials close to resting potential^18^. Calcium imaging studies on the interaction of neighbouring inputs^16^,^20^,^24^ have also pointed to a sublinear addition of neighbouring inputs. Inhibition persists for hundreds of milliseconds suggesting it is mediated by a muscarinic receptor for acetylcholine (mAChR), the neurotransmitter at the synapses onto the LGMDs^25^. And a neural network incorporating laterally spreading inhibition between unspecified retinotopic neurons on the input pathway into the input organization of the LGMD 1, reacted to looming objects and not to translating or receding ones^26^. However the likely site of this laterally spreading inhibition has been hard to pin down^27^. Synapses onto the LGMDs are cholinergic, activating calcium permeable nicotinic ACh receptors (nAChR) in the LGMD 1 and leading to its excitation^20^,^25^,^28^. These synaptic calcium signals reflect inputs which add together in a strongly sub-linear way in dendrites of the LGMD 1^16^,^17^,^24^,^29^. No systematic anatomical evidence exists for universal lateral connections between the neurons providing input to the LGMDs. In single sections, synaptic interconnections between inputs onto one of the LGMDs have been reported but from single sections it is not clear what the three dimensional arrangement of synapses is, or how common these are^18^,^25^. In this investigation we make 3-D reconstructions at different spatial scales from locusts of different species and ages, and establish definitive input connectomes for each LGMD. Input features shared by both collision sensing LGMDs will most likely be crucial to their selectivity for looming objects. We use a neural network simulating input to an LGMD to test the functional consequences of our findings.

## Results

### Identification and anatomical characterization of cells synapsing onto the LGMD 1

**Figure 1:**
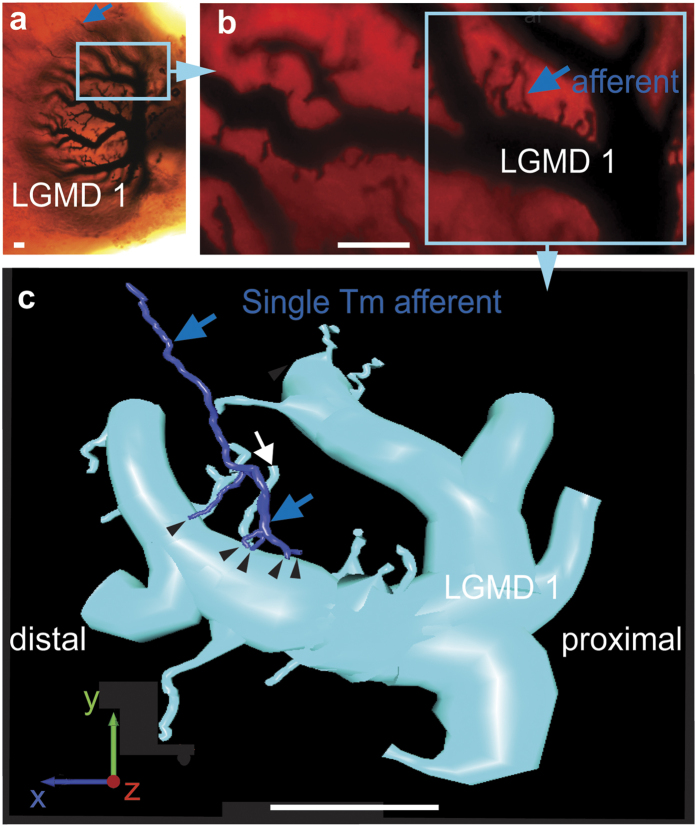
Trans-medullary afferent (TmA) axons contact LGMD 1 main dendritic field in the lobula. Scale bar is 10 μm in (**a**–**c**). (**a**) LGMD 1 in the lobula of the locust’s left optic lobe. The path the TmA axon takes from the medulla to LGMD 1 is arrowed. (**b**) The blue highlighted area in (**a**) is shown at higher magnification with the TmA axon’s termination on LGMD 1 arrowed. (**c**) A reconstruction of the area circled in (**b**) shows the TmA axon (blue arrows) terminating (black arrowheads) on LGMD 1. A white arrow indicates the LGMD 1 spine contacted by the TmA. Proximal is towards the body axis, distal is away from it. X, Y and Z are all defined according to body axes. X is directed dorso-ventrally, Y is directed proximo-distally and Z from anterior to posterior.

The anatomy of the cells providing input to the LGMDs, was revealed initially by intracellular staining (Fig. 1a,b) when dye injected into an LGMD 1 also revealed a presynaptic neuron. Four features identify it as a trans-medullary afferent cell (TmA), it has axon terminals in the outer lobula, its axon projects through the inner chiasm into the medulla where it branches and its cell body is located in a cortex of cell bodies adjacent to the medulla ((Fig. S1). The TmA terminal axon arborization in the lobula consisted of five processes that contact a 10 μm diameter LGMD 1 dendrite and a small, 0.5 μm diameter LGMD 1 spine (Fig. 1c).

**Figure 2:**
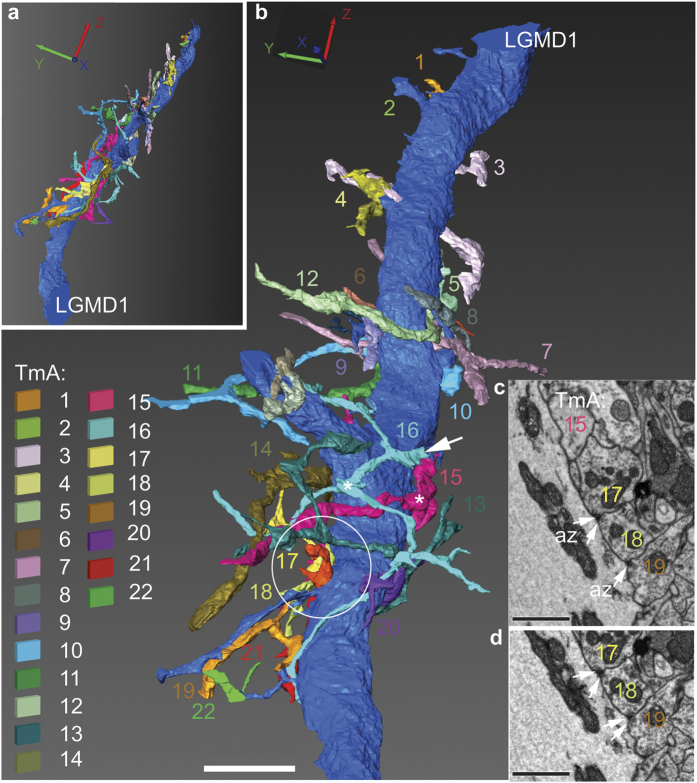
TmA axons synapse with a large LGMD 1 dendrite in a fourth instar *Locusta migratoria* hopper. (**a**) A single main LGMD 1 dendrite (cut obliquely) with synaptically connected TmAs. Individual TmA axons are identified by colour and number. TmA 2 is only visible in this view. (**b**) Detail from (**a**) to show TmA axons (1–22) make multiple connections at terminal boutons onto LGMD 1. A white arrow indicates the cut axon of TmA 16. Asterisks show dark pink TmA 15 and blue, TmA 16. A circle indicates the region of the reconstruction shown in (**c**,**d**). Scale bar is 6 μm. (**c**) SBEM image of synapses between TmAs and LGMD 1 from the reconstruction shown in (**a**,**b**). Synapses onto LGMD 1 at a terminal bouton are mostly reciprocal between two TmAs (two arrows TmA 17 and 18) or in the case shown here, only one TmA (TmA 19 single arrow) has an active zone (az) but the az in the second TmA starts in the next section (**d**). Scale bar is 1 μm. (**d**) SBEM image of synapses between TmAs and LGMD 1. Next section on from (**c**). Synapses onto LGMD 1 at a terminal bouton are now reciprocal between TmAs 17 and 18 and between TmAs 18 and 19. Scale bar is 1 μm.

To establish unequivocally that TmAs synapse with the LGMDs we used the ultrastructural evidence of synaptic contact provided by serial block-face-scanning electron microscopy (SBEM)^30^,^31^. We traced 22 TmA axons from their synapses with a 92 μm length of LGMD 1 dendrite, back toward the inner chiasm and the medulla (Fig. 2a,b and (Movie 1). The dendrite was cut obliquely with 562 serial sections making up the reconstruction, a total reconstructed distance of 22.48 μm across the dendrite (see methods for details). Four typical TmAs and their contacts are shown in two consecutive SBEM images from consecutive block-faces (Fig. 2c,d) used in the reconstruction in Fig. 2a. In section one, TmA cells 17 and 18 synapse reciprocally with the LGMD 1 and each other, with both TmA’s sharing a synaptic cleft and having dark presynaptic densities making up the synaptic active zone, (az, two arrows Fig. 2c). TmA 19 also makes a synapse onto TmA 18 (single arrow Fig. 2c) and in section two, TmA18 also synapses onto TmA 19 (two arrows Fig. 2d). These synapses are located on bouton-like regions of the TmA’s axonal branches. Although BSEM images allow serial reconstruction over large distances the images do not have the ultrastructural clarity of those made using the TEM, nor is it easy to follow the finest processes through dense neuropil, for this we turned to TEM.

### Connections between individual TmA cells as they synapse with the LGMDs

**Figure 3:**
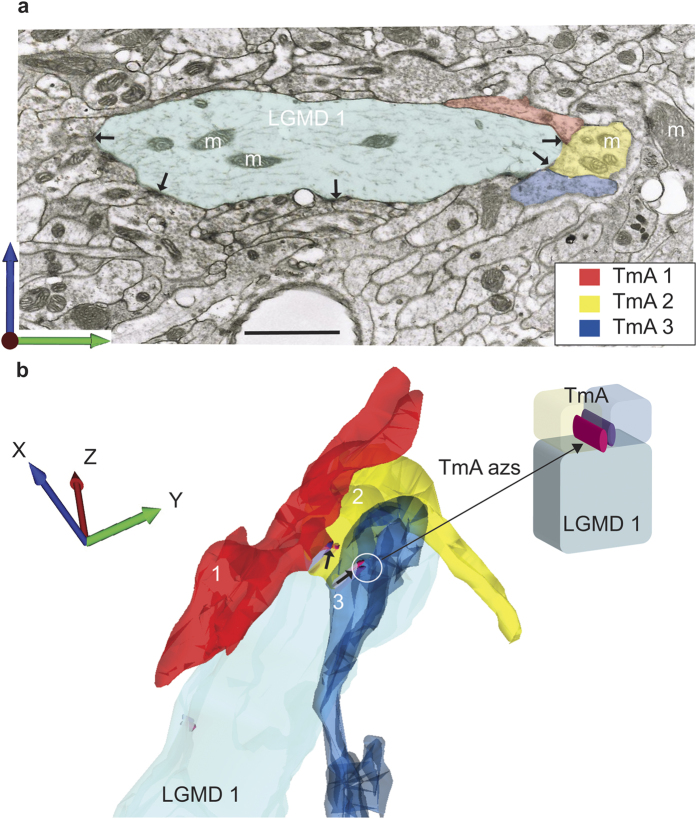
TmA to LGMD 1 connectome shows the prevalence of reciprocal TmA interconnections. In 20 consecutive TEM sections spanning a distance of 1.4 μm, every TmA (N = 62) and synapse (N = 118) with a 5 μm diameter LGMD 1 dendrite was traced. (**a**) First micrograph of the series with LGMD 1 (turquoise), three TmA terminal boutons (Tm 1–3) and their presynaptic az and those of other TmAs are indicated (arrows). Mitochondria (m) are visible both at TmA terminal boutons and in LGMD 1. Scale bar is 1.5 μm. (**b**) 3-D Reconstruction of Tm A1–3 drawn to the same scale as in (**a**). TmA terminals are shown semi-transparent so their relationships can be resolved. Azs are indicated by a crimson or purple rod. Reciprocal TmA synapses onto LGMD 1 have a common synaptic cleft and parallel azs.

**Figure 4:**
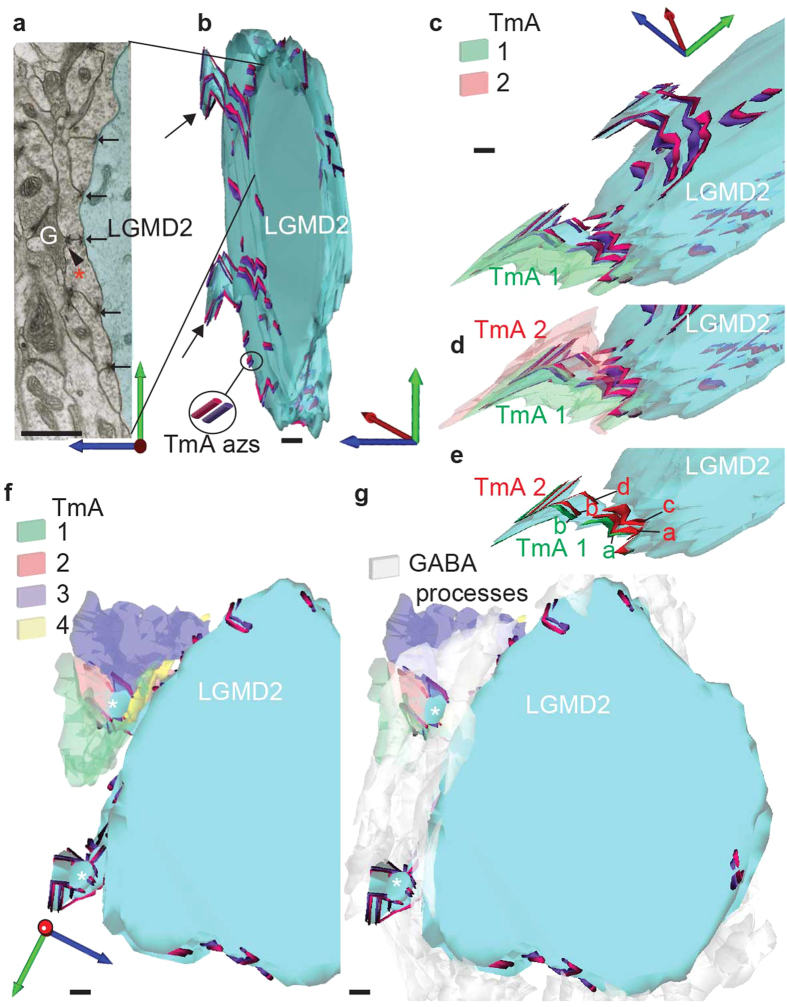
TmA to LGMD 2 connectome shows the prevalence of reciprocal TmA interconnections. In 20 consecutive TEM sections, spanning a distance of 1.4 μm, every TmA (N = 99) that synapsed (N = 122) onto a 12 μm diameter LGMD 2 dendrite was traced. In all cases synapses are made reciprocally between two TmAs, and with the LGMD 2. 17 GABA containing cells were also reconstructed. Scale bars are 1 μm. (**a**) Single section used for the reconstructions in (**b**–**g**). Reciprocal TmA synapses onto LGMD 2 have a symmetrical common synaptic cleft giving a V like cross-section (black arrows). Microcircuits (black arrowhead) were found between TmA terminals onto GABAergic cells (G) and from GABAergic cells back onto TmAs (not shown). (**b**) TmA azs are shown over the LGMD 2′s surface. All TmA synapses are made onto the LGMD 2, and reciprocally onto another TmA. (**c**) TmA 1 (green) makes and receives multiple synapses with other TmA cells and an LGMD 2 spine. (**d**) TmA 2 (red) tracks TmA 1 over the surface of the LGMD, both making synapses reciprocally. (**e**) TmA 1 (green) makes 2 output synapses (azs **a** and **b** in green) with TmA 2; while TmA 2 (red) makes four output synapses (azs **a**–**d** in red), two with TmA 1. (**f**) TmA terminals 1–4 synapse with neighbouring boutons around a dendritic spine of the LGMD 2 (asterisk), shown viewed from above in (**a**–**e**). (**g**) Numerous GABAergic terminals (white-gray profiles) surround the terminal TmA boutons and a dendritic spine of the LGMD 2, also shown in (**f**).

**Figure 5:**
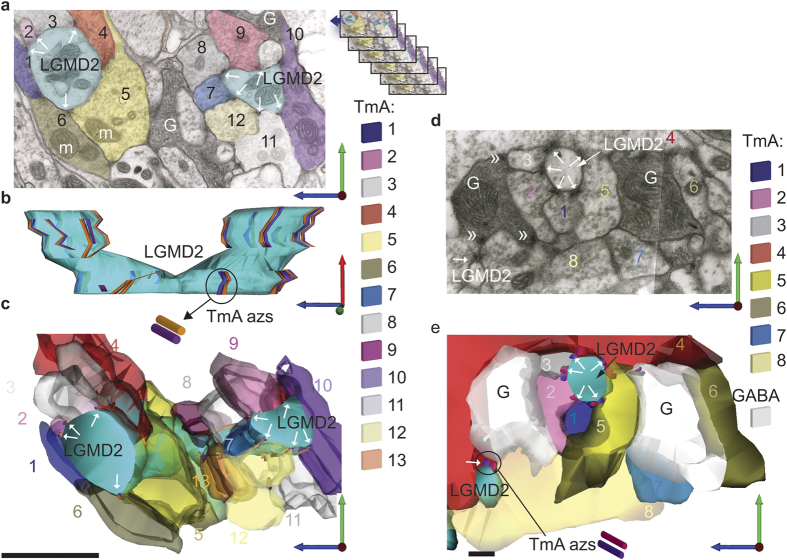
Reconstruction of TmA inputs onto the finest LGMD 2 dendrites. The reconstructions were taken from a region of lobula adjoining that shown in Fig. 4. (**a**) TEM section, from the start of the reconstruction shown in (**b**). Twelve of the TmAs that were reconstructed are present in this section, each TmA is represented by a different colour, as indicated in the key. The series used for (**b**) is shown in the inset. Azs with vesicles attached, mark each synapse (white arrows indicate reciprocal pairs of synapses). GABAergic cells (G) are seen closely associated with TmA numbers 7, 9–12. m: mitochondrion. (**b**) Reconstruction of TmA inputs to a fine LGMD 2 dendrite. Presynaptic azs extend over the surface of a bifurcating LGMD 2 process. Repeatedly, azs of two TmA cells run in parallel over LGMD 2. Azs are shown as orange or purple cylinders. In this view the TmA profiles themselves have been hidden. (**c**) Reconstruction of the LGMD 2 process and TmAs shown in (**a**,**b**). Terminal boutons of TmAs1–13 surround the LGMD 2 dendrite (blue-green) making synapses onto it (white arrows indicate pairs of reciprocal synapses). Colouring of each TmA is as in (**a**). GABAergic cells are not shown. (**d**) TEM section, from the start of the reconstruction shown in (**e**). TmA terminal boutons surround the 0.5 μm diameter LGMD 2 dendrite, synapsing with it and with each other (white arrows). GABAergic cells (G) with their dark cytoplasm and variable, flattened vesicles contain three electron-dense presynaptic azs making output synapses (double chevrons) onto TmAs labelled 2, 3 and 4 plus several unreconstructed TmAs. (**e**) Reconstruction of a TmA connectome surrounding the finest LGMD 2 process shown in section in (**d**). Azs are shown as pink or purple cylinders. Terminal boutons of TmA 1–8 (shown above) surround the small LGMD 2 dendrite (blue-green) making synapses onto it (white arrows indicate pairs of reciprocal synapses). GABAergic cells are reconstructed in pale grey but their synapses are not shown. Scale bar is 1 μm in (**a**–**c**) and 200 nm in (**d**,**e**).

**Figure 6:**
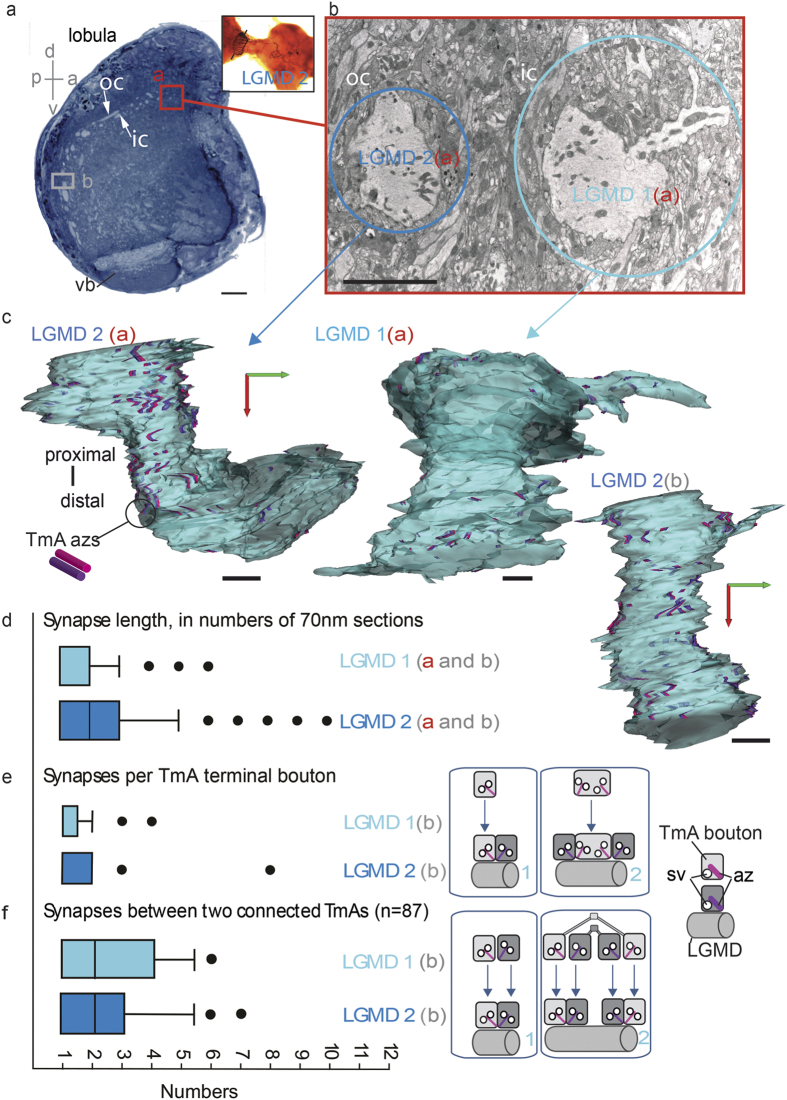
Quantification of TmA inputs over matched lengths of LGMD 1 and 2 dendrites. (**a**) Section through the lobula region of the optic lobe, taken at the start of the LGMD reconstruction. Dendrites of LGMD 1 and 2 cut in cross-section occur in two crescents, an inner and outer crescent respectively, in the outer region of the lobula complex. The inset shows an intracellularly stained LGMD 2 with the level of the section indicated by a dotted line. Two dendrites from each of the LGMD 1 and 2 were analyzed. The two regions the dendrites were traced from are indicated by boxes: “a” and “b”. oc: outer crescent; ic: inner crescent; vb: ventral bundle of axons. Scale bar is 10 μm. (**b**) Higher power TEM image showing LGMD 1 and 2 dendrites at “a”. LGMD 1 has a long bifurcating branch which is easily recognized at the top of its reconstruction in (**c**). Scale bar is 5 μm. (**c**) All TmA synapses onto LGMD 1 and 2 at “a” and LGMD 2 at “a” and “b”. Reconstructions all have the same orientation. Scale bar is 2 μm. (**d**) Comparison of the length of the presynaptic TmA azs of LGMD 1 and 2, sampled at “a” and “b”. Az length was measured by calculating the number of 70 nm sections the az extended through. Box and Whisker plot showing median, ±50% and +75% range. Values outside the 75% range are shown as filled circles. See results section for a statistical analysis of this data. (**e**) Number of output synapses made by each individual LGMD 1 and 2 TmA terminal bouton in region “b”. Inset shows the example of one, or two output synapses per bouton. Az: active zone; sv: synaptic vesicle. (**f**) Number of times two connected TmAs in region “b”, synapse with one another. Inset illustrates the case of one, versus two synapses per connected TmAs.

To reveal the connectome of multiple TmAs and LGMDs at high resolution, we reconstructed all synaptic inputs of a volume of dendrite from each LGMD through 20 consecutive serial TEM sections (Figs 3 and 4) or through shorter series for very fine processes (Fig. 5). The series were taken from LGMD 1 and LGMD 2 dendrites from position “a” in the lobula, indicated on Fig. 6a,b. In this region there were large sized LGMD dendrites, their profiles identifiable because they were arranged in an inner and outer semi-circle corresponding to LGMD 1 and 2 dendrites respectively^18^,^25^ ((Fig. S2). We were motivated by the fact that shared features of the LGMDs input connectomes will be crucial for their common selectivity for looming objects. First we concentrated on the LGMD 1. Three typical TmA cells and their synapses are shown, labelled in the first micrograph of the series (Fig. 3a) and then followed through the subsequent reconstruction (Fig. 3b). Synaptic azs occur between each of the three neighbouring TmAs, one in each of the outer TmAs, and two in the middle TmA (black arrows). This same pattern of reciprocal connections between TmAs as they contact the LGMD was found in the inputs onto the LGMD 2 regardless of the size of the dendrite (Figs 4a–g and 5a–e). Individual micrographs from the start of the LGMD 2 series clearly show that TmA synaptic azs occur between pairs of neighbouring TmAs and both the neighbouring TmA and the LGMD are postsynaptic targets (black arrows in Fig. 4a and white arrows in Fig 5a,d). However, uniquely in the LGMD 2, TmA cells contacting the LGMD 2, also synapse (black arrowhead in Fig. 4a) with γ-aminobutyric acid (GABA) containing processes (cells marked “G” in Figs 4a and 5a,d), identified by their darker cytoplasm and variously shaped flattened synaptic vesicles^18^. It is also clear from the individual micrographs that these GABA containing neurons also synapse back onto TmA cells (double chevrons in Fig. 5d). When the TmA to LGMD 2 connectome was reconstructed and all the presynaptic azs mapped over the surface of the LGMD 2, the azs form parallel double lines extending through multiple sections (TmA azs Figs 4b–e and 5b,c,e). The two parallel lines reflect the continuing reciprocal arrangement of the synapses between neighbouring TmAs and can be seen most clearly when the LGMD 2 is seen in side view (Fig. 5b). All synapses onto adult LGMD 2 have this same reciprocal arrangement. Even the finest diameter LGMD 1 and 2 dendritic spines (1–2 μm across; Fig. 4) emerging from larger diameter branches, and small terminal dendrites (0.4 μm, Fig. 5d,e) receive numerous TmA inputs. For example, in Fig. 4c–f when boutons of selected TmA cells contacting a short LGMD 2 spine (TmA 1 and 2) were revealed along with their presynaptic azs we can see a bouton can make more than one synapse with a bouton of a neighbouring TmA as well as with the LGMD 2. This relationship between TmAs is quantified in a subsequent section of the results. In this and all other LGMD 2 reconstructions we found GABA containing processes surrounding the boutons of the TmA cells (white processes in Figs 4g and 5c). GABA circuitry was unique to the LGMD 2 and not found in LGMD 1 processes in the same region.

### TmA synapse length and LGMD synaptic density

To assess if the pattern of connections seen at the finest scale in the previous section persisted over a wider scale we compared both LGMDs from the same 90 TEM sections through the lobula (neuropil regions “a” and “b” Fig. 6a). In both these regions there were large sized LGMD dendrites, which we could identify because they were arranged in an inner and outer semi-circle corresponding to LGMD 1 and 2 dendrites respectively ((Fig. S2 and Fig. 6b shown for region “a” only). TmA inputs onto the LGMD 1 and 2 were measured, counted and their density calculated. In total, the LGMD 1 received 297 inputs in region “a” and 622 in region “b” and the LGMD 2 received 335 synapses in region “a” and 515 synapses in region “b”. Those synapses spanning more than two sections are shown in the reconstructions of the LGMD 1 and 2 (Fig. 6c) and clearly reveal that, within the bounds of human error, all TmA synapses were arranged reciprocally, with two azs in parallel. Input to the main dendritic fan of both the mature LGMD 1 and 2 are exclusively in this form. Synaptic azs were different lengths in the two LGMDs (Fig. 6c,d), they were significantly shorter in the LGMD 1 (region “a” Mann-Whitney U = 34725.5, P = <0.001; region “b” U = 122962, P = <0.001). Mean synapse lengths were 155 nm (±SEM 5.6) for the LGMD 2, but only 107 nm (±SEM 4.9) for the LGMD 1. From these reconstructions we found that the LGMD 1 had a mean dendritic diameter of 6.5 μm ± 2.3 SEM at “a” and 7.5 μm ± 2.5 SEM at “b”, while LGMD 2 had a mean dendritic diameter of 5 μm ± 0.5 SEM at “a” and 10 μm ± 2.5 SEM at “b”. Combining the value for mean dendritic diameter, with synapse number for each neuron in each region, we calculated an input density per μm^2^ of 1.95 in the LGMD 1 at “a” and 3.15 at “b”. Corresponding values for the LGMD 2 were 2.54 and 2.21. These figures then allowed us to calculate the mean-synapse-length per μm^2^ for each LGMD. Giving to an upper bound to mean-synapse-length per μm^2^ for the LGMD 2 of 0.4 μm, and 0.34 μm for the LGMD 1.

### Quantifying patterns of TmA synaptic connectivity

From the 90 sections we selected a series of 20 perfect sections with no gaps or folds and we recorded 87 TmA cells synapsing with LGMD 1 or LGMD 2 dendrites, over a 1.4 × 10 × 7 μm volume (from region “b” shown graphically in Figs 3 and 4), and analyzed their connections (Fig. 6e,f). TmA axons divided into 2 to 5 axon terminals, each with a bouton, making output synapses on to LGMDs (inset Fig. 6e). The median number of synapses made per TmA bouton was 1 (Fig. 6e), a Mann-Whitney U Test showed no significant difference between LGMDs (Mann-Whitney U_29,39_ = 543, P = 0.724). The median number of synapses per two connected TmAs was 2 (Fig. 6f) irrespective of which LGMD the bouton was connecting with, a Mann-Whitney U Test showed no significant difference between LGMDs (U_42, 45_ = 871, P = 0.52). In summary, this means that the usual condition is, each TmA connects with multiple TmAs with one synapse per connection. Each of the 2–5 boutons contacting a median of two other TmAs. We tested this overall connectivity experimentally by counting the number of synapses per bouton, boutons per TmA and number of TmAs in contact with a LGMD 1 or 2 dendrite. The data was available in Reconstruct as part of our tracing and labelling of TmA processes through the 20 serial TEM sections. To establish how many separate, individual TmA cells are in synaptic contact we calculated a connectivity matrix. Median connectivity in the matrix was 1.5 for the LGMD 1 and, 2 for the LGMD 2 and a Mann-Whitney U test showed these differences were not significant (U_43,44 _= 752 P = 0.08). Highest connectivity was seen in the LGMD 2 where two TmAs each paired with six other TmAs. In the LGMD 1 the highest connectivity was four, one TmA paired with four other TmAs. This estimate is a lower bound to connectivity as the area reconstructed may have included incomplete TmA outputs.

### Calculating overall TmA to LGMD 1 and 2 synapse numbers and density

**Figure 7:**
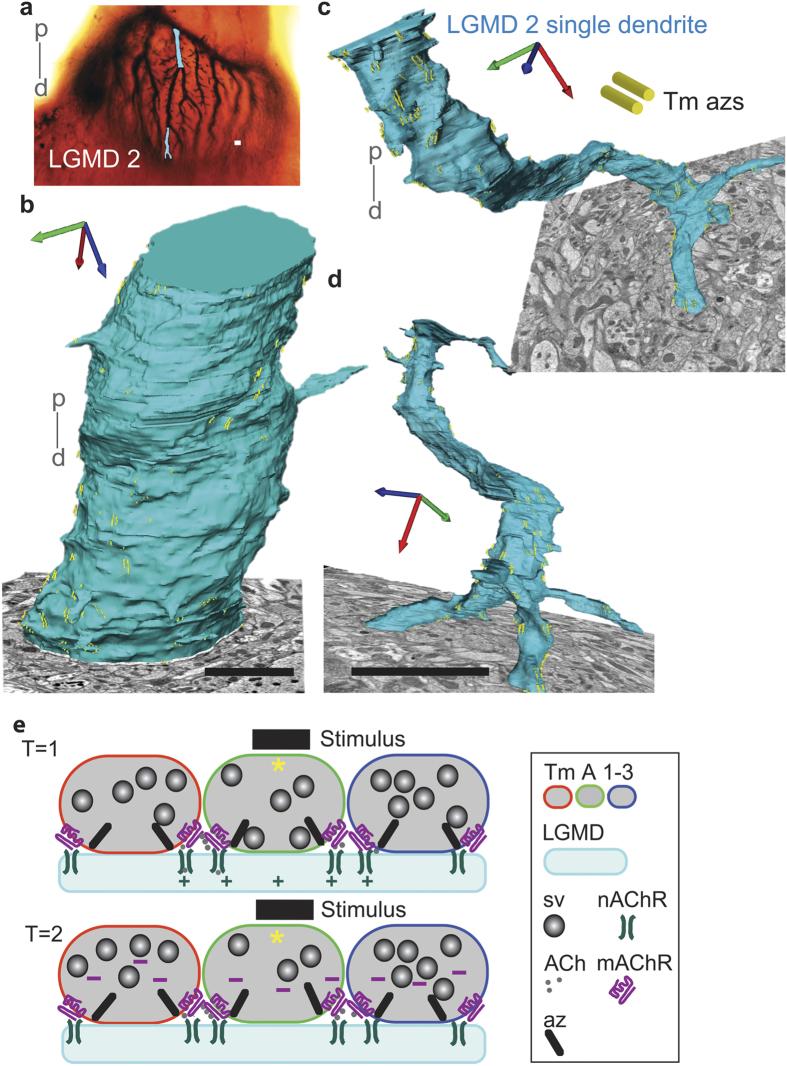
Wide-scale quantification of TmA synaptic inputs onto a main LGMD 2 dendrite. Consequences of the interconnections between TmAs for the selective response of both LGMDs to looming stimuli. (**a**) SBEM was used to reconstruct the inputs to two 16.8 μm lengths of a single, large LGMD 2 dendrite. The area reconstructed is indicated in blue, on a stained LGMD 2 neuron from the lobula of an adult locust. P, proximal; d, distal. Scale bar is 5 μm. (**b**) Reconstruction of the proximal section of a LGMD 2 dendrite, with the SBEM image from which it was traced. Presynaptic azs, in yellow are paired and occur with a density of 0.24/μm^2^ with 6.9 synapses/μm of dendritic length. 116 synapses were detected over the 16.7 μm dendritic length. Scale bar is 5 μm. (**c**,**d**) Reconstructions of the branched, distal section of the LGMD 2 dendrite, shown in (**b**). Perspective has been used to enhance the 3-D projection of the neuron but this means the scale bar (5 μm) only refers to where the bar is situated. For reference the trifurcation spanned 7 μm. 235 synapses were detected over the 16.7 μm length of the dendrite. In the reconstruction presynaptic azs, in yellow were paired and occur with a density of 1.04/μm^2^ with 14.05 synapses/μm. (**e**) Three LGMD 1 or 2 TmA terminal boutons linked with each other and a LGMD, at reciprocal synapses. The two panels represent successive times during a loom. The object moves over the receptive fields of three TmAs (asterisks). The degree of LGMD excitation (+), following activation of each TmA, is indicated by the size of the +. Two TmA boutons are connected by reciprocal synapses so as well as being presynaptic to the LGMD, each TmA is both presynaptic and postsynaptic to the other. Consequently TmAs have receptors for their own neurotransmitter, and when excited, cause lateral inhibition in their neighbours (L-inhibition via the neighbours’ mAChRs) and in themselves (S-inhibition via their own mAChRs). ACh, acetyl choline; Az, active zone; mAChR, muscarinic Acetylcholine receptors; nAChR, nicotinic Acetylcholine receptors; Sv, synaptic vesicle.

LGMDs have extensive arborizations in the outer lobula over which approach of a moving object is detected^5^,^6^,^32^. To interpret the functional significance of the overall synaptic input for looming sensitivity, we assessed total numbers of synapses, their density, and their number per facet, making use of the greater spatial reach provided by SBEM. We reconstructed two 16.8 μm lengths from proximal and distal terminal ends of a single LGMD 2 dendrite in an adult *Schistocerca gregaria* locust (Fig. 7a–d, (Movies 2 and (3). Each length consisted of 240 serial sections. The thicker, more proximal LGMD 2 dendrite (Fig. 7b) had 116 synapses, with a synapse density of 0.24/μm^2^ or 6.9 synapses/μm dendrite length, the distal dendrite (Fig. 7c,d) despite its smaller diameter had 235 synapses at a density of 1.04/μm^2^ and 14.05 synapses/μm dendrite length. To calculate total length and total surface area of dendrites in adult LGMD 1 and 2 and so arrive at the total numbers of synapses for each LGMD we applied a growth factor of 11% (derived from the percentage increase in facet number in the compound eye between the last larval instar and adult^7^) to the data gained from reconstructions for both LGMDs in last instar locusts, the largest sized locust for which the data is available in Neuroexplorer^TM^ 7,8. Growth between instars is not uniform over the entire LGMD, rather 806 facets are added at the anterior margin of the compound eye and to accommodate input from these new facets new LGMD 1 and 2 segments are added to the finer, distal-most dendrites of less than 7 μm for the LGMD 2 and less than 5 μm for the smaller LGMD 1. Applying the 11% growth to just these dendrites in a dataset of all dendrite lengths, surface areas and diameters available in Neuroexplorer^TM^ from reconstructed 5^th^ instar LGMDs^7^,^8^, gives an estimate of a total adult LGMD 1 dendritic length of 12.1 mm and a total LGMD 2 dendritic length of 13.7 mm. And applying the 11% growth to LGMD surface area ((Table S1) gives a total dendritic surface area for the adult LGMD 1 of 61,712 μm^2^ and for the LGMD 2 of 75,629 μm^2^ ((Table S1). Now, applying our measured synapse densities, per μm^2^ to dendrites of appropriate diameter allows us to arrive at a total synapse number for the adult LGMD 1 of 131,000, and for the adult LGMD 2 of 186,000 ((Table S1).

### Inter-connections sharpen selective responses to looming, compared with near-miss, or translating stimuli

The consequence of the high density reciprocal dyadic synapses of both TmA to TmA, and, TmA to LGMD, is that two TmA boutons face each other across a synaptic cleft: both are pre and postsynaptic to the other (shown schematically in Fig. 7e). They will each have receptors for their own neurotransmitter, and on excitation, pass inhibition laterally to their neighbours (L-inhibition) and to themselves (S-inhibition).

**Figure 8:**
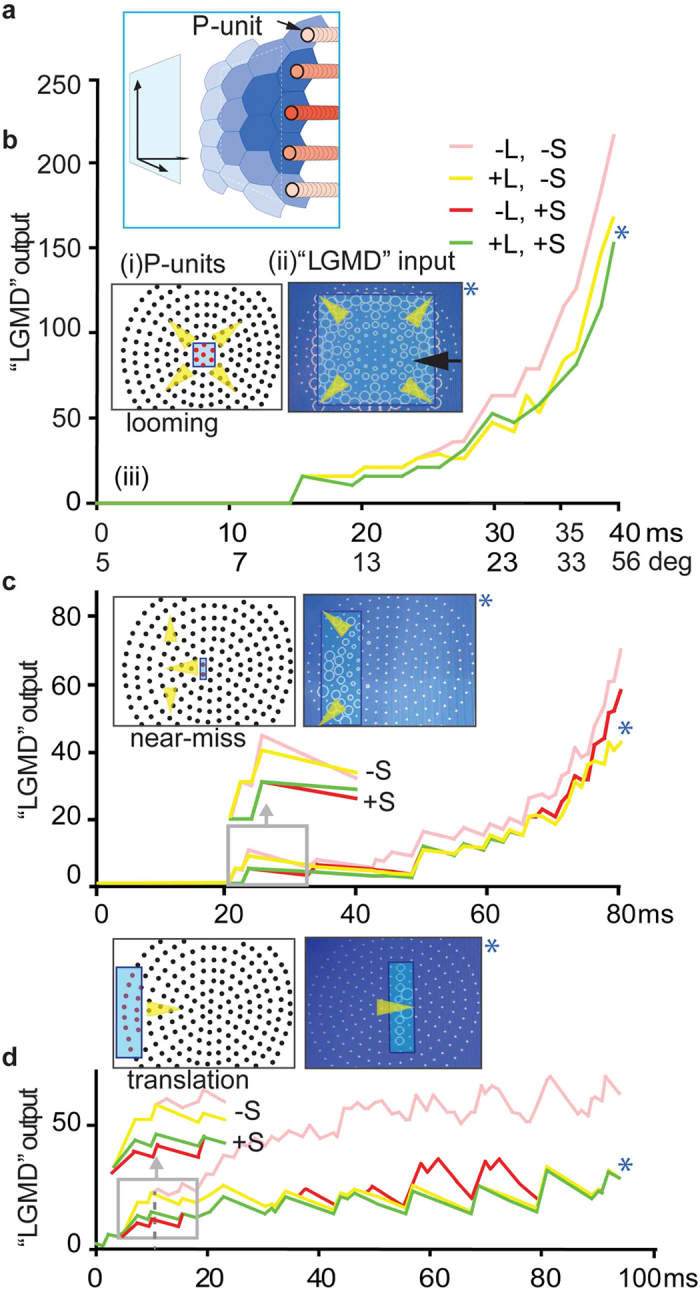
A dynamic neural network approach to explore the functional consequences of TmA reciprocal interconnectivity for the selective response of the LGMDs to looming. (**a**) Schematic illustration showing the visual stimulus (looming object) mapped onto the photoreceptor-lamina monopolar array (P-units) which provides retinotopic input onto the simulated looming detector (details of neural network given in methods). (**b**) Looming object (details of stimulus given in methods). (i) P-units: Initial stimulus position (blue outline) on P-units with stimulated P-units shown in red, and the direction of stimulus image movement is indicated by yellow arrow-heads. (ii) “LGMD” input: The final snapshot of local input to a LGMD dendrite (“LGMD” input), with L- and S-inhibition. Gaps in local “LGMD” input dendrite activation (black arrow) can be seen where L- and S-inhibition prevented activation during the early stages of the loom. Eventually as collision nears, all units which the edge passes over are fully activated (circle of maximal diameter). In the locust this would lead to the strong, accelerating spiking in the LGMD 1 needed to trigger escape in flight^50^. (iii) Graph: “LGMD”-unit output during the loom under four conditions: with no nearest neighbour inhibition (pink); nearest neighbour L-inhibition and no S-inhibition (yellow); S-inhibition but no L-inhibition (red) or both L- and S-inhibition (green). The red and pink lines are superimposed and only the pink is shown. A blue asterisk indicates when on the graph the “LGMD” input snapshot was taken. Object angular subtense is also shown on the graph during the 40 ms loom. (**c**) Near-miss object trajectory. Rectangular object (details of stimulus given in methods) approaching, but passing to one side of the centre of the array. Layout and labelling as in (**b**). (**d**) Translating object. Rectangular object (details of stimulus given in methods) moving across the input array at a fixed distance from it. Layout and labelling as in (**b**).

To uncover the functional consequence of this arrangement we used the dynamic neural network approach we had developed^26^. We chose this approach because the neural network already implemented lateral connections between retinotopic inputs on the pathway to the LGMD, although we had to adapt it for this investigation to incorporate the precise details of the interconnections among the TmAs themselves which was not known at the time the neural network was originally published. The network is described in the methods section and consisted of a hexagonal array of P (photoreceptor)-units (Fig. 8a), onto which we mapped successive images of translating, near-miss or approaching objects, one image per simulated millisecond. Retinotopy was preserved with summation of local inputs on dendrites of the “LGMD” unit. For this investigation we modified the extent and the gain of the lateral inhibition (L-inhibition) from that described in ref. 26. The strength of L-inhibition was increased by 12% from^26^ to 200 × 1/6% of activation and the spread was restricted to the nearest 6 neighbouring units in the hexagonal array. For the first time S-inhibition was included in the neural network. Local excitation finally converged to provide output of the “LGMD”-unit. In Fig. 8b–d the mapping of the stimulus onto the P-units, and the local excitation of the LGMD 1 dendrite are shown for simulated object approach (Fig. 8b), near-miss (Fig. 8c) and translation (Fig. 8d). The final output of the neural network, representing the excitation of the main dendritic fan of the LGMD 1 as a whole, was then compared during looming (Fig. 8b bottom panel), near-miss (Fig. 8c bottom panel) and object translation (Fig. 8d bottom panel). We plotted “LGMD” output under four conditions: with no S- or L-inhibition (pink lines); with L-inhibition only (yellow lines); with S-inhibition only (red lines) or with both L- and S-inhibition (green lines). With no inhibition the response of the neural network to a looming object outstripped the response to a translating object, even when the image of the translating object is a similar height to the final image height of the looming object (pink lines, bottom panel, Fig. 8b,d). L-inhibition alone, cut back the response to all three stimuli (Fig. 8b–d), but its effect was greatest during translation (Fig. 8d) where the simulated LGMD output was halved in final size. With L-inhibition alone, the neural network predicted that the effect (divergence of pink and yellow lines Fig. 8b–d) begins 12 ms after motion starts for the translating stimulus, and, 25 ms after motion begins for the looming stimulus. At this time during the looming stimulus, gaps occur in the pattern of local excitatory “LGMD” input and units remain as dots due to their lack of excitation (arrow Fig. 8b “LGMD” input). When S-inhibition is present (red and green lines Fig. 8b–d), the suppressive effect on “LGMD” output begins earlier than with L-inhibition, 5 ms after motion begins for the translating object and 20 ms for the near-miss. For a looming object, S-inhibition alone had no effect, and, the pink and red lines are superimposed in the graph in Fig. 8b, with only the pink shown. However combining S and L-inhibition (green line) led to a change in “LGMD” output when compared to L-inhibition alone (yellow line) suggesting, in the case of looming, an interaction between the two types of inhibition.

For non-collision courses S-inhibition had its greatest effect at the beginning of object motion (compare pink and yellow lines with red and green Fig. 8c,d). We found the maximum suppressive effect of S-inhibition on “LGMD” output when inhibition persisted for 7 ms, as in the simulations shown, when inhibition persisted for less than 7 ms the suppressive effect was proportionally reduced over the range from 7–3 ms. Increases in S-inhibition persistence beyond 7 ms had no additional effect under the conditions tested. This time course for inhibition is within the range found in the locust ocellar visual system, where an IPSP begins 4–5 ms after a presynaptic cholinergic neuron starts to depolarize from its resting potential, and the time-to-peak is 7 ms^33^. The effect of S-inhibition was dependent on the shape and structure of the simulated object, and the velocity of object motion.

Previously, physiological investigations have not pointed to a role for L-inhibition at the level of the inputs to the main dendritic fan of the LGMD^20^,^34^,^35^ even though strong lateral inhibition is a feature of the overall pathway^18^,^22^,^23^ but this study suggests L- and S-inhibition are universal features of the direct inputs to both LGMDs and shows how L- and S-inhibition could have an unsuspected role in generating selectivity for approaching objects.

## Discussion

The cells that provide input to the main dendritic fan of both LGMDs (1 and 2) are small TmA cells from the medulla. TmAs don’t simply excite the LGMDs, they also connect with other TmAs. A feature of the connections in all TmAs was they were made reciprocally with another TmA, regardless of the size of LGMD branch or what species or age the locust was. TmAs that contact the main dendritic fans of the LGMD 1 or 2 are probably a single class of neurons as, so far all their axons entered the chiasm linking the medulla and lobula, but confirmation of this must await serial reconstructions of the dendrites of individual TmAs in the medulla. A neural network helps separate the contributions of the interconnections to the preferential LGMD response to looming.

TmA to LGMD synapses on fine dendrites of the LGMDs are densely packed: with 2–3.1 synapses per μm^2^ dendritic surface (0.5–0.32 μm^2^ per synapse) in the LGMD 1, and 2.2–2.6 per μm^2^ dendritic surface (0.46–0.39 μm^2^ per synapse) in the LGMD 2. Vertebrate grey matter synapses, for comparison generally have a spacing of 6 μm^36^, with a measured postsynaptic hippocampal shaft membrane area, for example of 0.66 μm^2^ per synapse^37^. LGMD synapse packing can be denser than *Drosophila* photoreceptors which were thought to be at the upper limit of packing density^38^. Combining our data with published measurements ((Table S1) allowed us to arrive at a total synapse number for the adult LGMD 1 of 131,000, and for the adult LGMD 2 of 186,000 ((Table S1). The total adult LGMD 2 input is comparable to the number of input synapses from parallel fiber synapses onto Purkinje cells in rat cerebellum^39^. The estimate, based on reconstructions of silver stained LGMDs, may still be an underestimate because the finest LGMD spines and dendrites are the most difficult to trace using silver staining. Using published data, we can also understand the proportion of each LGMD devoted to processing information from a single facet.

By dividing LGMD 1 and 2 dendritic surface areas by total number of facets in the adult (8134, ref. 7), we get a per-facet-area of 7.6 μm^2^ for the LGMD 1 and 9.3 μm^2^ for the LGMD 2 and per-facet-synapse-numbers of 131,000/8134 = 16 for the LGMD 1 and 186,000/8134 = 23 for the LGMD 2. Similar to the maximum number of connections made by each TmA as estimated by the connectivity matrix method. The connectivity matrix gave a maximum per-TmA-connectivity for the LGMD 1 of 24 (each TmA was connected with 4 others with a maximum 6 synapses per connection) and a maximum per-TmA-connectivity for the LGMD 2 of 42 (each TmA was connected with 6 others with a maximum 7 synapses per connection). The general conclusion from comparisons of per-facet-connectivity with per-TmA-connectivity is that there is one TmA per facet for each LGMD with all of the six neighbouring facets interconnected in the LGMD 2 and a subset in the LGMD 1.

In insects, synapses are commonly divergent (dyadic), with one presynaptic cell and two different post-synaptic cells^40^. TmA to LGMD synapses are cholinergic^18^,^25^ which means that TmA terminals release ACh into the synaptic cleft, where it binds to receptors of two post-synaptic cells, the LGMD and a neighbouring TmA. On the LGMDs ACh receptors open fast-acting ionotropic channels leading to calcium entry and excitation^16^. When two nearby small patches of retina are stimulated with motion simultaneously, responses in LGMD, measured over 250 ms, sum together in a strongly sub linear way, with the mean response to both stimuli being close to the first stimulus alone^16^,^29^ a result that is compatible with a simple driving force effect where activation of two inputs results in a postsynaptic voltage closer to the reversal potential of the synapses, as seen in hippocampal pyramidal cells^41^ but is also compatible with suppressive effect of presynaptic inhibition extending laterally from one stimulated TmA to its neighbour. Muscarinic ACh receptors on TmA boutons, when stimulated could account for the presynaptic lateral inhibition lasting tens of milliseconds that is a feature of LGMD 1 responses recorded electrophysiologically^18^,^42^. Indeed, inhibitory muscarinic receptors for ACh, (A-type muscarinic cholinoceptor or mAchR-A [CG4356]), occur in the *Drosophila* Trans-medullary afferent cell, T5^43^ and throughout the arthropods^44^. Electrophysiological and pharmacological support for muscarinic presynaptic inhibition in insect sensory pathways comes from the cockroach cercal to giant-wind-sensitive-interneuron synapse, where single cercal hair mechanoreceptors release ACh which both excites the target postsynaptic giant wind-sensitive neuron via nAChR and inhibits the same mechanoreceptor via inhibitory presynaptic mAChRs^45^,^46^. But ultrastructural evidence is still required before interposed inhibitory GABAergic neurons with mAChRs can be ruled out. GABAergic neurons with mAChRs, were found between the wing stretch receptor and a flight motoneuron that depresses the wing in the locust^47^. In the case of the LGMD 1 no GABA immunoreactive cells were interposed between the TmA boutons and their synapses with the LGMD 1. And even in the case of the LGMD 2, TmAs synapsed directly with the LGMD 2 as well as synapsing with GABA immunoreactive cells and in turn receiving input from them (Figs 4 and 5).

Figure 7e illustrates schematically how the ubiquitous, high density, reciprocal, dyadic synapses between the TmAs and the LGMDs would work: two TmA boutons face each other across a synaptic cleft, both are pre and postsynaptic to the other. When a dark edge moves into its receptive field the excited TmA depolarizes, ACh is released at its presynaptic az. ACh binds to ionotropic nicotinic receptors on the LGMD dendrite and to slower inhibitory muscarinic receptors on the neighbouring bouton (L-inhibition) and on the active bouton itself (S-inhibition).

Although both the LGMD 1 and 2 are looming detectors, the LGMD 2 is different from the LGMD 1 because of the involvement of GABAergic neurons in the TmA input circuitry. LGMD 2 responses are also distinct: it spikes at rest, shows no excitatory response to stimuli that are lighter than the background^6^ and it may be more involved in the hiding responses of the locust that are triggered early in the loom^8^ but the way LGMD 2 input circuitry shapes these properties is not yet understood.

Locusts are not amenable to genetic manipulations in a way common in *Drosophila*, however using a different approach we can dissect the relative contributions of L- and S-inhibition. We used a dynamic simulation of the LGMD 1 pathway and uncovered a role for the proposed S-inhibition in the selective responses to looming over translating objects. We found the strongest effect of L- and S-inhibition at the beginning of translation, where the moving, leading edge of an object leaves a trail of self-inhibition which the trailing edge moves into, the S-inhibition reduces the response to the trailing edge. In the simulation a velocity of 0.75 m/s was tested. At the beginning of translation in particular, S-inhibition was more effective than L-inhibition at reducing LGMD output. During object approach in our simulation, both L- and S-inhibition are overcome during object approach by excitation, when the image reaches a subtense of 33 degrees and the output of the LGMD output rises steeply after this until the end of the loom. A feature of looming is acceleration of the image edge motion as collision nears^5^,^48^,^49^, neural latencies of individual excitatory inputs shorten as a consequence of this acceleration, further synchronizing the arrival of excitatory inputs onto the LGMD 1^20^. Although this feature is not explicitly included in the current simulation, where latencies were fixed, it would further increase LGMD output to looming stimuli as collision approached and may shorten latencies of both L- and S-inhibition as well. In the locust, this would lead to strong accelerating spiking in the LGMD 1 which is needed to trigger escape in flight^50^. A behavioural threshold to looming often occurs when an object’s image subtends around 33 degrees on the eye, range 15–40 degrees: locust^11^,^50^,^51^ *Drosophila*^52^, the pigeon^53^, frog^54^, fish^55^ and the cat^56^. The locust LGMD 1 is a model system for single neuron computation^14^,^16^ and now the computations performed over the surface of both LGMDs by the many synapses tuned to the image-changes that signal a looming object, provide a framework to understand more of the computations underlying looming detection.

## Materials and Methods

Adult, fourth and fifth instar locusts of the species *Locusta migratoria* and *Schistocerca gregaria* were taken from our crowded colony.

### Light microscopy

To reveal the anatomy of single afferent neurons and the LGMD 1 and 2, in adult locusts (*Locusta migratoria*) intracellular injection of hexamminecobaltic ions was used^57^. The structure of the LGMD 1 and 2 (not shown) and individual TmA cells were then reconstructed from the whole mounted optic lobe using Neurolucida^TM^ (MBF Bioscience, Williston, Vermont, USA). Silver staining of the LGMD 1 and 2 in a fifth instar *Locusta* as described in^8^, allowed us to reconstruct both LGMDs to measure total dendritic length and surface area using data in Neuroexplorer^TM^ (MBF Bioscience, Williston, Vermont, USA).

### Serial Block Face Scanning Electron Microscopy (SBEM) and Transmission Electron Microscopy (TEM)

For TEM, optic lobes of adult *Locusta migratoria* were fixed for 3 hours in 2% glutaraldehyde, 2% formaldehyde in 0.1 M sodium phosphate buffer, pH 7.4, rinsed in buffer, post fixed in 2% osmium tetroxide in buffer for 1 hour, rinsed, dehydrated in a series of graded ethanols and embedded in resin (TAAB). During dehydration the blocks were block contrasted with 2% uranyl acetate dissolved in 70% ethanol. TEM micrographs were then taken of the LGMDs in the lobula region of adult locusts. The lobula was identified with reference to a series of 1 μm, toluidine blue stained cross sections taken at 5 μm intervals through the lobula complex. When the level containing many LGMD 1 and 2 profiles in a crescent, was reached (Figs S2 and 6a) we made series of 70 nm thick sections using a Diatome^TM^ diamond knife. Sections were placed on washer grids, two to three per grid, stained with lead citrate, and viewed using a Philips transmission electron microscope. Intracellular staining and serial sectioning of the LGMD 1 and 2 in the optic lobe show each is identifiable by its location in the distal lobula and its position relative to the other, the LGMD 2 being closer to the posterior surface of the optic lobe^25^ as illustrated in (Figure S2. Initially we confirmed we were able to follow fine branches of the LGMDs and recognize them in successive sections. After finding we could recognize even the finest processes we made a 90 section series through the lobula that we used for our reconstructions. Unless otherwise stated sections and reconstructions were from adult locusts. SBEM with a section thickness of 70 nm can underestimate synapse numbers because active zones (azs) are at the limits of its resolution and they were only identified if they persisted through two or more block faces. By contrast those in the TEM could be identified in a single section but the number of serial sections could not approach those possible with SBEM.

We used the higher resolution and flexibility afforded by the Philips CM100 TEM to focus repeatedly on different regions in each section, looking at low and high magnification at many individual LGMD dendrites to confidently trace the same processes in successive sections. A disadvantage over SBEM was that each of the 90 sections expanded to a different extent when it was flattened prior to being collected on a grid which gives the reconstructions of individual dendrites a less smooth appearance. 3-D reconstructions were made using Reconstruct software^58^ available free from http://www.bu.edu/neural/Reconstruct.html. In this analysis the LGMD 1 and 2 were compared from the same sections in adjacent areas of neuropil and both had undergone the same fixation regime. Sections were aligned using features such as trachea and mitochondria that had stable positions through a number of sections.

For SBEM, we used optic lobes from the wingless fourth instar *Locusta migratoria* hoppers and adult *Schistocerca gregaria*. Input synapses were reconstructed for one dendrite of the hopper LGMD 1, and synapse numbers and arrangement, were reconstructed for two segments of one dendrite of the adult LGMD 2. To enhance contrast within the blocks, a modified version of the *en bloc* contrast protocol was used^59^. After fixation for 3 hours in 2.5% glutaraldehyde, 2% formaldehyde in 0.1 M sodium cacodylate buffer, pH 7.4 the optic lobes were rinsed with the same buffer and post fixed in 1% osmium tetroxide (reduced with potassium hexacyanoferrate (II) mixed with cacodylate buffer) for 1 hour, rinsed, put into an contrast enhancer, thiocarbohydrazide 10% (0.1 g/10 ml) for 1 hour, rinsed, put into 1% osmium tetroxide for 30 min, rinsed, placed into 1% aqueous uranyl acetate for 2 hours at 60 °C, rinsed, placed into Walton’s lead aspartic staining solution with a pH of 5.5 overnight, rinsed and dehydrated in a series if graded ethanols and embedded in resin (TAAB, hard mixture). Both blocks were cut using a 3View ultra-microtome (Gatan), mounted in the chamber of an ESEM Quanta 600 FEG environmental scanning electron microscope (FEI). Images of the respective block faces were recorded at electron energy of 3 kV for the hopper and 4 kV for the adult. The voxel size was 10 × 10 × 40 nm^3^ for the hopper and 10 × 10 × 70 nm^3^ for the adult. In one block for the adult the cutting thickness was checked afterwards using mitochondria to calculate section thickness which did not deviate more than 3%. Section thickness of the first block, from a fourth instar locust, *Locusta migratoria*, was 40 nm. 562 serial sections were taken; a total distance of 22.48 μm. The proximal and the distal part of one LGMD 2 dendrite were cut from the second block at 70 nm thickness, over a distance of 16.8 μm for each part. Section thickness of the second block, from an adult locust, *Schistocerca gregaria* was 73.7 nm covering a total distance of 33.6 μm.

### Identifying Synapses with SBEM

Synapses on serial block-face-scanning electron micrographs (SBEM) are identifiable because an electron dense presynaptic bar, the az reaches into the presynaptic cell^60^; the bar and its surrounding vesicles are visible as a darkening in the cytoplasm of the presynaptic cell. Previously we have shown that SBEM micrographs can allow the identification of synapses with the same accuracy as in TEM micrographs^61^. For reliability we only included synapses if at least two postsynaptic neurons were visible and the presynaptic cell’s darkening stretched over two or more sections. Our TEM studies showed a number of synapses only visible in one 70 nm section which may introduce a bias against very short synapses. The 3-D reconstruction was done with the software AMIRA v 5.4.2 (FEI Visualization Sciences group). For this purpose, the acquired image stacks were transformed into single micrographs using Digital Micrograph v2.11.1404.0 (Gatan), median filtered and converted to gray scale. jpg images with IrfanView v4.28. The images were then loaded into AMIRA™.

### Dynamic simulation

Microcircuits that were consistently found were tested for their contribution to the selective response of the LGMD 1 to looming objects by incorporating them into a neural network-LGMD based on^26^. Input to the network was provided by images of approaching or translating objects at one image per simulated millisecond. The network consisted of layers of neurons. The first layer represented the retina and lamina, and consisted of hexagonally arranged P-units, onto which the visual stimulus was mapped. Each P-unit was a combined photoreceptor and lamina monopolar (L) neuron with one P-unit per ommatidium. The inter-P-unit angle was set to 3.3 degrees, which is 33–50% greater than that observed in the locust but was used to spread the field of view in the 250 simulated P-units. The second layer, representing the small-field, retinotopic, TmA input onto the LGMD 1, segregated the excitatory and inhibitory processes, each generated from the same brief pulse of excitation by the photoreceptor unit. The excitation from a unit in the second layer passed directly to a local “LGMD” input unit in a corresponding retinotopic position in the third layer, a unit that represented the input to a small local segment of LGMD 1 dendrite. The inhibition, if present, was spread to the maximum number of “LGMD” input units revealed anatomically in this investigation. “LGMD” input units also showed self-inhibition (S-inhibition), newly coded in the neural network as a refractory period of 7 ms at the combined inhibitory and excitatory input to the LGMD. In this study two types of motion were simulated, both with and without S- and L-inhibition. The output of the LGMD unit was compared to each motion type. The first motion was translation in which a 70 × 20 mm rectangle moved 5 m across the input array, 100 mm away at 0.8 m/s taking 90 ms, without approaching (a velocity of 370°/s), the second was looming, where a 75 × 75 mm square approached at 10 m/s over from a distance of 400 mm from 500 mm (*l/v* = 3.75 ms for a circular object 75 mm in diameter). The locust LGMD responds directionally to looming stimuli approaching at a wide range of velocities, from 0.3–10 m/s^5^ and to translation ≥272 down to 4°/s^5^,^49^,^62^.

## Additional Information

**How to cite this article**: Rind, F. C. *et al*. Two identified looming detectors in the locust: ubiquitous lateral connections among their inputs contribute to selective responses to looming objects. *Sci. Rep.* **6**, 35525; doi: 10.1038/srep35525 (2016).

## Supplementary Material

Supplementary Information

Supplementary Movie S1

Supplementary Movie S2

Supplementary Movie S3
