# Supplementary material for: Two identified looming detectors in the locust: ubiquitous lateral connections among their inputs contribute to selective responses to looming objects
Source: Sci Rep. 2016 Oct 24;6:35525. doi: 10.1038/srep35525 (PMC5075876; doi:10.1038/srep35525)
Supplement: Supplementary Information [file srep35525-s1.pdf]

Supplementary Material: Two identified looming detectors in the locust: ubiquitous lateral connections among their inputs contribute to selective responses to looming objects.

F. Claire Rind*[1], Stefan Wernitznig[1,2], Peter Pölt[4,5], Armin Zankel[4,5], Daniel Gütl[2], Julieta Sztarker[1,6] , and Gerd Leitinger**[2,3,].

Figure S1 Trans-medullary afferent (TmA) axons project in the inner optic chiasm from the LGMD 1 main dendritic field in the lobula to the medulla. Scale bar is 10 µm in (a) and (b).

(a) An LGMD 1 in the lobula of the locust's left optic lobe, viewed from the back looking forward. In this view the dendrites of the LGMD 1 in the lobula are closest to the back surface of the lobe. The path the TmA axon takes from the LGMD 1 to the medulla is arrowed. The cell body is out of the plane of focus at the front of the optic lobe. Inset shows an enlarged section of (a) including the TmA axon

(b) An LGMD 1 in the lobula of the locust's left optic lobe, viewed from the front showing the cell body of a TmA afferent. The cell body is small, about 2µm in diameter and is located in a cortex of similarly sized cell bodies adjacent to the medulla neuropil. Inset shows an enlarged section of (b) including the TmA cell body.

Figure S2 Large sized LGMD dendrites and their profiles are identifiable in the optic lobe because they are arranged in an inner and outer semi-circle corresponding to LGMD 1 and 2 dendrites respectively. No other processes of this size occur in this position. This enabled clear identification of dendrites as belonging to either the LGMD 1 or 2. In this investigation we serially sectioned the lobula region of the optic lobe and used the single large strap-like trunk of the LGMD 2 dendritic tree to identify it. The branches of the LGMD 2 dendritic tree, then emerged from this trunk in later more distal sections. This section showing the LGMD 2 trunk was taken 10µm before, and proximal to, the LGMD reconstruction shown in Figure 6. Inset is of an intracellularly stained LGMD 2 from another preparation with the level of the section shown by a dotted line. vb: ventral bundle of axons. Scale bar is 10 µm.

Video S1 Video made using Amira^{TM} of a single main LGMD 1 dendrite (cut obliquely) with synaptically connected TmAs. Individual TmA axons are identified by color and number. See Figure 2 legend for details.

Video S2 Reconstruction showing all TmA synaptic inputs onto the thickest basal part of a large finger-like LGMD 2 dendrite, each TmA az is indicated as a yellow tube on the surface of the LGMD 2. The last SBEM image from the end of the 16.8 µm reconstruction is also shown. See Figure 7 for more details. This video was made using Amira^{TM}. Amira has problems putting one

continuous skin over a material when too few pixels overlap between one section and the next, so Amira™ can break up the synapses into several parts, although these were scored as one synapse. This happened during the reconstructing and smoothing process.

Video S3 Reconstruction showing all TmA synaptic inputs onto the thinner part of a large finger-like LGMD 2 dendrite, each TmA az is indicated as a yellow tube on the surface of the LGMD 2. The last SBEM image from the end of the 16.8 µm reconstruction is also shown. See Figure 7 for more details. The video was made using Amira™.

Table S1 Total input synapse numbers and densities of the adult LGMD 1 and 2 main dendritic tree. Data for the LGMD 1 and 2 derived from the TEM and BSEM reconstructions of individual dendrites in adult locusts (Figs. 3-7) combined with data from the total branching pattern, dendrite diameter and surface area of the LGMD 1 and 2 from reconstructed 5th instar locusts[8]. Synaptic densities for the largest LGMD 1 and 2 diameter dendrites were taken from SBEM data from the LGMD 2* (Fig. 7). Three categories of branches in the 5th instar were distinguished by their diameters then in order to derive the adult values for total surface area and synapse numbers, 11% growth[7] was applied to only the smallest branch category. We did this because at a moult new ommatidia are only added at the

anterior margin of the eye and that area is represented on the finest most anterior LGMD dendrites (≤4.9 for the LGMD 1 and ≤6.9 for the proportionally larger LGMD 2), which add new segments at each moult[8]. *Data taken from the LGMD 2 as comparable data is not yet available for the LGMD 1.

**LGMD 1**				
Segment mean diameter (µm)	≤4.9	5-7.9	≥8	All
5th INSTAR				
Surface area (µm^2)	46,015	9,628	1,008	56,650
ADULT				
Surface area (µm^2)	51,076	9,628	1,008	61,712
Synaptic density/ µm^2	1.96^{TEM}	3.18^{TEM}	0.24^{SBEM*}	-
Synapse no.	100,109	30,617	242	130,968
**LGMD 2**				
Segment mean diameter (µm)	≤6.9	7- 9.9	≥10	All
5th INSTAR				
Surface area (µm^2)	60,645	5,190	3,123	68,958
ADULT	67,316	5,190	3,123	75,629
Surface area (µm^2)				
Synaptic density/ µm^2	2.58^{TEM}	2.23^{TEM}	0.24^{SBEM}	
Synapse no.	173,675	11,574	752	186,000

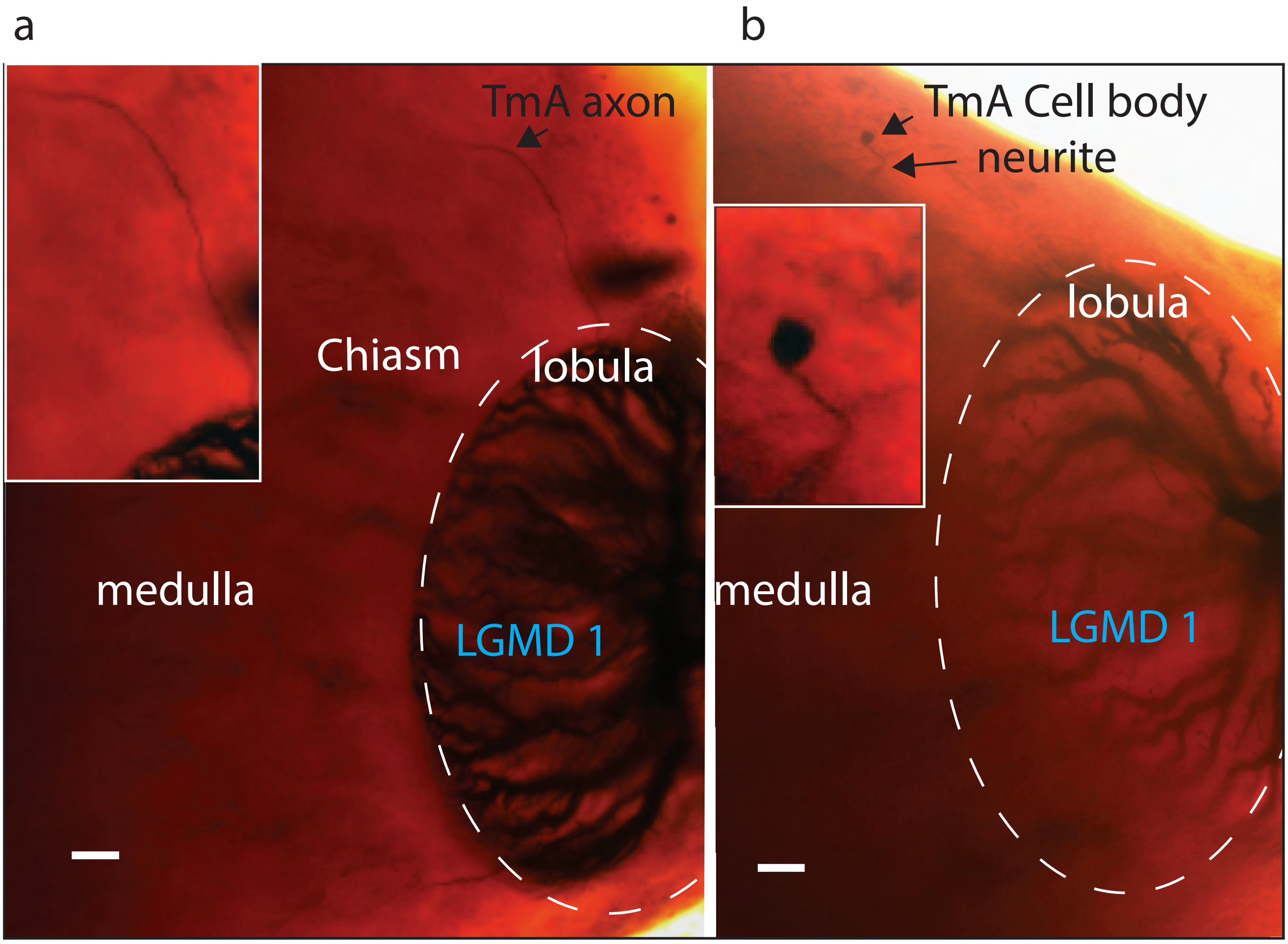
a
b
TmA axon
Chiasm
lobula
medulla
LGMD 1
TmA Cell body
neurite
lobula
medulla
LGMD 1

Figure S1

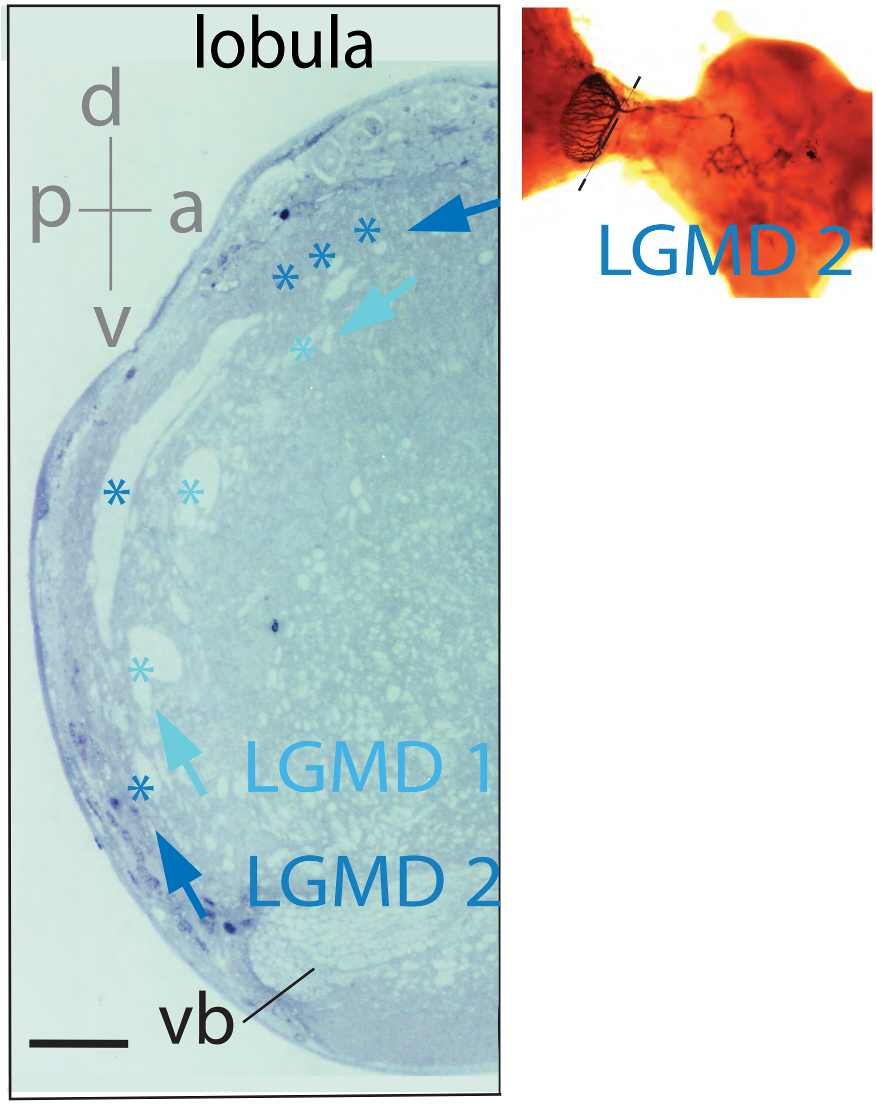

Figure S2
